# Patterns of help‐seeking for mental health problems in 1001 self‐identified neurodivergent adolescents who self‐harm

**DOI:** 10.1002/jcv2.70050

**Published:** 2025-10-07

**Authors:** Simona Skripkauskaite, Galit Geulayov, the OxWell Study Team, Mina Fazel, Rohan Borschmann

**Affiliations:** ^1^ Department of Psychiatry University of Oxford Oxford UK; ^2^ Department of Experimental Psychology University of Oxford Oxford UK; ^3^ Centre for Adolescent Health Murdoch Children's Research Institute Royal Children's Hospital Melbourne Victoria Australia; ^4^ Justice Health Group School of Population Health Curtin University Perth Western Australia Australia; ^5^ Oxford Health NHS Foundation Trust Oxfordshire UK

**Keywords:** adolescent, help‐seeking, mental health, neurodivergence, self‐harm, services

## Abstract

**Background:**

The prevalence of self‐harm is high across neurodivergence. Help‐seeking is an important component of the management of mental health problems and self‐harm, but it is low in adolescents who self‐harm. Combined with a common neurodivergent challenge of encountering multiple barriers in accessing support, it is unclear whether and how neurodivergent adolescents who self‐harm seek, receive, and perceive mental health support in comparison to their peers.

**Method:**

Participants were 12,209 adolescents (aged 11–18 years) from the OxWell 2023 Student Survey in English schools. Of these, 1001 (8.2%) self‐identified as neurodivergent (dyslexic, dyspraxic, autistic, and/or have attention deficit hyperactivity disorder) and self‐reported self‐harm. We conducted three separate mixed‐effect models to examine (1) help‐seeking, (2) receipt, and (3) perceived helpfulness of the support.

**Results:**

Proportionally more adolescents who reported both self‐harm and self‐identified neurodivergence sought help compared to their peers who reported no self‐harm and/or no neurodivergence. In line with previous studies, all adolescents were more likely to seek support from informal than formal sources and least likely to seek support online. Yet, self‐identified neurodivergent adolescents who self‐harm were more likely to seek formal support than their peers. When support was sought, adolescents in all groups were likely to receive it. However, those who reported either self‐harm or self‐identified neurodivergence were less likely to perceive the support received as helpful, especially from formal services.

**Conclusion:**

Self‐identified neurodivergent adolescents who self‐harm report greater unmet need for mental health support, but not due to a lack of help‐seeking. These findings, instead, point to potential issues with the acceptability of support received. This highlights the need to better understand which forms and locations of support are most acceptable to meet the specific needs of this population.

## Key Points

**What's known?**

- Many adolescents who self‐harm do not seek any help afterwards and those who do mostly utilise informal sources, such as friends and parents.

**What's new?**

- Self‐identified neurodivergent adolescents who self‐harm are more likely to seek support from informal than formal sources and least likely to seek support online.
- But they are more likely to seek formal (pastoral school staff or mental health services) support compared to their peers.
- Adolescents who self‐report self‐harm or neurodivergence are not necessarily refused support but are less likely to perceive it as helpful, especially from formal sources.

**What's relevant?**

- These findings highlight that both neurodivergent adolescents and those who self‐harm merit special consideration in the design and evaluation of mental health interventions and services.

## Graphical abstract

Adolescents who self‐harm are also more likely to seek support from informal than formal sources and least likely to seek support online. But neurodivergent adolescents who self‐harm are more likely to seek any and especially formal (pastoral school staff or mental health services) support than their peers. Adolescents who self‐report self‐harm or neurodivergence are not necessarily refused the support but are less likely to perceive it as helpful, especially from formal sources.

## INTRODUCTION

Neurodiversity refers to the natural variation in human neurological phenotypes and, in turn, our experiences of the world (Fletcher‐Watson, 2022). It is grounded in a social model of disability, which suggests that ‘disability’ occurs as a result of a poor person‐environment fit, such as inaccessible social or support infrastructures, rather than as a result of within person characteristics (den Houting, 2019; Dwyer, 2022; Pellicano & den Houting, 2022). Neurodivergence is an umbrella term defining a heterogeneous group of individuals who have a neurocognitive profile that diverges from the majority (Fletcher‐Watson, 2022). It has been estimated that around 15%–20% of the population may be neurodivergent (Doyle, 2020; Maciver et al., 2023), which includes a range of neurodevelopmental variations such as autism, attention deficit hyperactivity disorder (ADHD), dyslexia, or dyspraxia. While there are specific challenges and strengths associated with each individual neurotype (Dwyer, 2022), there is an increased recognition of overlapping traits and behaviours (Apperly et al., 2024), frequent co‐occurrence (Astle et al., 2022; Brimo et al., 2021), and persistent issues with misdiagnosis or delayed diagnosis (Chellappa, 2024). In the United Kingdom (UK), these diagnostic challenges are compounded by substantial increases in numbers being referred to child and adolescent mental health services (CAMHS), leading to a strain in many services with long waiting times as specific training and substantial time is often needed for clinicians and services to be able to make a formal diagnosis. Strict diagnostic criteria further compound timely access to assessment and support for neurodevelopmental differences (Autistica, 2024; Children's Commissioner, 2025). As such, a transdiagnostic approach that centres on individual lived experience and self‐identified support needs, rather than rigid diagnostic categories, may offer a more inclusive and effective framework for understanding and addressing shared challenges within the neurodivergent community. While the accuracy and clinical validity of self‐identification remains contested (see Fellowes, 2024), emerging evidence indicates that both diagnosed and self‐identified neurodivergent adults have similar rates of internal stigma, quality of life, self‐esteem, and mental health outcomes, all of which are significantly worse than non‐neurodivergent comparison groups (Kroll et al., 2024, 2025; McDonald, 2020).

Self‐harm is disproportionately prevalent across neurodivergence (Oliphant et al., 2020). Self‐harm is defined as ‘an intentional act of self‐poisoning or self‐injury, irrespective of the motivation or apparent purpose of the act’ (NICE, 2022). Self‐harm during adolescence is a risk factor for numerous adverse clinical and social outcomes (Borschmann et al., 2017), including suicide (Hawton et al., 2020). A 2021 meta‐analysis of 31 studies found pooled odds of self‐harm that were more than three times higher in autistic than non‐autistic children and adults (Blanchard et al., 2021). Autistic people are also significantly more likely to die by suicide than those in the general population (Hirvikoski et al., 2016; Hwang et al., 2019; Kirby et al., 2019). Similarly, higher proportions of children with ADHD than without ADHD present to healthcare services following self‐harm (DiScala et al., 1998; Schott et al., 2022) and have a history of suicide attempts (Sultan et al., 2021). Whilst the links between other neurodivergence umbrella categories (e.g., dyslexia or dyspraxia) and self‐harm have received less research attention, it has been suggested that dyslexic individuals may also be more likely to use self‐harm as a coping mechanism for emotional distress (Alexander‐Passe, 2015).

Help‐seeking for mental health difficulties is broadly defined as the process by which individuals actively seek external assistance to cope with mental health concerns (Rickwood & Thomas, 2012). This process encompasses both formal (e.g., mental health professionals or school and community resources) and informal (e.g., friends, family) sources (Barker et al., 2005; Rickwood & Thomas, 2012), and increasingly online help‐seeking due to added anonymity and accessibility (Pretorius et al., 2019). Active help‐seeking among adolescents can be understood through the Common‐Sense Model of Self‐Regulation and established help‐seeking frameworks, which together highlight the dynamic and adaptive interplay of cognitive, emotional, and social processes that shape how young people recognize, interpret, and respond to mental health difficulties (e.g., Brooks et al., 2022; Hagger & Orbell, 2022; Leventhal et al., 2016). The help‐seeking framework delineates this process into several stages: problem recognition, expression of the need for support, identification and evaluation of potential sources of help, and deciding whether to engage with them (Rickwood et al., 2005). Young people often prefer informal support rather than accessing professional help (Barker et al., 2005; Rickwood et al., 2005).

For adolescents who self‐harm, these processes are often complicated by heightened self‐stigma, ambivalence about the efficacy of professional support, and a strong sense of personal responsibility to manage distress alone (Rowe et al., 2014). As a result, the overwhelming majority of adolescent self‐harm does not come to formal clinical attention (Geulayov et al., 2018). Many adolescents who self‐harm do not seek *any* help afterwards and those who do, also, mostly utilise informal sources, such as friends and parents (Geulayov et al., 2022). Research shows that previous home and community‐based care can offer protective benefits against later emergency hospitalization for autistic children and young people (Mandell et al., 2019), while inability to access sufficient support is associated with likelihood of planned or attempted suicide in autistic adults (Moseley et al., 2025).

Little is known about how experiences of seeking and receiving support for mental health problems in neurodivergent adolescents who self‐harm compared to the experiences of their peers (both neurotypical and neurodivergent) who do not self‐harm. Autistic adults and parents of autistic children report encountering multiple barriers in identifying and accessing acceptable health care support (for all health needs, including mental health care) (Crane et al., 2019; Jackson et al., 2020; Vogan et al., 2017), despite seeking such support more often (Weiss et al., 2018; Zerbo et al., 2019). Children and young people with ADHD also report unmet needs regarding mental health problems (Vijverberg et al., 2020). Furthermore, the existing evidence suggests that autistic people may prefer online or computer‐based communication to foster new supportive relationships (Burke et al., 2010; Gillespie‐Lynch et al., 2014). Autistic adults have previously ranked communication via email, text, and instant messaging as preferred modes of communication for access to services (Howard & Sedgewick, 2021). Another qualitative study with autistic and ADHD adolescents and adults revealed that, in addition to the opportunity to communicate in writing, online support was also perceived as beneficial due to it being accessible in one's own home and perceived as immediate (Sehlin et al., 2018). Taken together, this suggests that neurodivergent adolescents who self‐harm may differ from their peers in both the sources from which they seek mental health support, and in the perceived availability and helpfulness of the support.

Adolescent help‐seeking for mental health concerns occurs within a complex context shaped by both cultural norms and structural formal service limitations, therefore it is important to situate help‐seeking behaviours within the broader service landscape in England. Formal care is largely delivered through the National Health Service (NHS), specifically CAMHS, which are free at the point of access. However, certain services in CAMHS can have long waiting times to access, specific eligibility thresholds, and regional disparities in provision (Children's Commissioner, 2025). While many schools provide pastoral support or access to mental health professionals and para‐professionals, the scope, staffing levels and consistency of this provision vary considerably between local authorities, reflecting disparities in investment, implementation strategies, and local partnerships (see Fazel & Soneson, 2023). Furthermore, although school enrolment is high among 11 to 18‐year‐olds in England, a small minority (2%–3%) are not enroled or severely persistently absent (missing more than 50% of school sessions), with higher risk among those with severe mental health problems or other vulnerabilities (Long & Roberts, 2025); of note, data from this group are not collected in the OxWell Survey data presented here. For some families, particularly those in rural or socioeconomically disadvantaged contexts, additional barriers such as transportation, time off work, or navigating complex referral pathways may further restrict access (Allwood, 2020; Reardon et al., 2017). Stigma around mental health in the UK remains a significant barrier for child and adolescent formal help‐seeking (Aguirre Velasco et al., 2020; Radez et al., 2021). These structural and interpersonal challenges are likely particularly pronounced for neurodivergent adolescents and those who self‐harm.

### Present study

Given the high overlap between neurodivergence and self‐harm, as well as low rates of help‐seeking in adolescents who self‐harm, and reported barriers to support in neurodivergence, it is crucial to understand whether, where, and how neurodivergent adolescents are seeking mental health support and how this interacts with their self‐harm behaviours. This study aimed to investigate: (1) whether self‐identified neurodivergent adolescents who self‐harm differ in their patterns of help‐seeking from those who do not self‐harm, (2) whether they are more or less likely to receive the support they seek, and (3) how helpful they perceive that support to be. We explored these questions across formal, informal, and online sources of support, and compared adolescents who (a) self‐harm; (b) self‐identify as neurodivergent; (c) both, or (d) neither.

Based on previous literature, we hypothesised that help‐seeking on average will be more pronounced in adolescents who self‐harm than those who do not, but that those who self‐harm will be more likely to seek help from informal (e.g., parents, siblings and friends) than formal (e.g., CAMHS or Educational Mental Health Practitioner) or online (e.g., online friends, website, or helpline) sources in comparison to those who do not self‐harm. When sought, informal support will be offered more often and perceived as more helpful than formal support, but this will not differ between those who self‐harm and those who do not. We further hypothesised that help‐seeking, on average, will also be more pronounced in adolescents who self‐identify as neurodivergent than those who do not and that self‐identified neurodivergent adolescents will be more likely to seek help from formal and online than informal sources in comparison to neurotypical adolescents. When sought, online support will be received more often and perceived as more helpful than formal support by self‐identified neurodivergent in comparison to neurotypical adolescents. We did not a priori expect group differences in helpfulness of informal support between self‐identified neurodivergent adolescents and their peers. Due to the limited literature on the help‐seeking in neurodivergent individuals who self‐harm, we did not propose any a priori hypothesis for the interaction between self‐harm, self‐identified neurodivergence, and help‐seeking, receipt of, or perceived helpfulness of the support.

## MATERIALS AND METHOD

### Participants and procedure

Participants were adolescents aged 11–18 years who took part in the OxWell 2023 Student Survey. The OxWell Survey is a repeated cross‐sectional survey of schools and further education colleges in England, in which students self‐report on a range of questions relating to their mental health and wellbeing, life experiences, and behaviours (Mansfield et al., 2021). The OxWell Survey uses a school‐based, convenience (rather than probabilistic) sampling strategy that recruits students through state and independent schools, as well as Further Education Colleges, across several local authorities covering a range of socioeconomic areas. Participating schools are primarily recruited directly by local authorities, who enrol participants on the basis of either (1) a parental opt‐out model with student assent (for under 16‐year‐olds); or (2) students' informed consent (for those 16‐years and older). The data analysed here were collected from February to March 2023 from adolescents in school years 7 to 13 (corresponding to ages 11–18 years). Participation in the OxWell survey was voluntary, and participants did not receive any incentives to take part. The exact day of survey administration was arranged by each school and was not systematically announced to students in advance. While participants were drawn from multiple regions, they may not represent the broader population of 11–18‐year‐olds in England, particularly those not in school due to illness, disability, or disengagement.

In total, 28,702 eligible students, based on predefined inclusion criteria (Skripkauskaite et al., 2023), completed the 2023 OxWell survey for years 7 to 13. Of these, 9116 (31.8%) were excluded due to missing information on their self‐harm history. Another 3953 (13.8%) and 994 (3.5%) participants were excluded due to missing information on neurodivergence and help‐seeking, respectively. Finally, 690 (2.4%) participants provided conflicting responses, such as simultaneously stating that they sought support from multiple sources and that they did not seek any support from any sources and thus were excluded from the final sample (for sample characteristics at each stage of attrition see Supporting Information S1: Table S1). This resulted in the final sample of 12,209 participants from 78 schools, primarily across Berkshire, Liverpool, Milton Keynes and Oxfordshire.

### Measures

All questions in the OxWell Student Survey are reviewed and iteratively refined with input from stakeholders including service commissioners and young people (see Acknowledgements), aiming to ensure age‐appropriate wording.

#### Self‐identified neurodivergence

Participants were asked ‘Do you consider yourself to be dyslexic/dyspraxic, and/or autistic, and/or have ADHD (i.e., neurodivergent)?’ with response options of: ‘Yes’, ‘No’, ‘Not Sure’, ‘Prefer not to say’. Participants received no further definition of the different types of neurodivergence. The variable was dummy coded into ‘Yes’ (1) and ‘No’ (0) categories with the rest of the answers (‘Not Sure’ and ‘Prefer not to say’) treated as missing.

#### Self‐harm

Participants' self‐harm history was primarily ascertained based on their free‐text descriptions, supported by their responses to two structured items, in line with a self‐harm ascertainment method that was developed and used in the CASE study (Madge et al., 2008). They were first asked ‘Have you ever deliberately self‐harmed (for example by taking an overdose or deliberately injuring yourself on purpose in some other way)?’ They were provided with response categories of: ‘Yes’, ‘No’, ‘Prefer not to say’, ‘Not sure what this means’. Those who indicated they had self‐harmed, were asked a follow‐up multiple‐choice question ‘In what ways have you self‐harmed? (tick all that apply)’ with response categories of: ‘I injured myself on purpose (e.g., by cutting myself)’ or ‘I took an overdose on purpose’. They were then invited to provide a free‐text response to describe their act, that is, ‘Please can you tell us what you did?’. This free‐text response served as the primary source for verifying whether self‐harm had occurred. Two researchers (G.G. and R.F.N.) independently screened these responses to determine whether they described a deliberate act of self‐harm. Disagreement between G.G. and R.F.N. were discussed and resolved with a senior clinician (M.F.) to ensure clinical accuracy. For example, responses indicating accidental injury (e.g., ‘I accidently fell’) were recoded as not self‐harm, whereas those indicating reasons rather than method (e.g., ‘I was upset about…’) were treated as missing, as information was insufficient to determine if the person had self‐harmed. Their response patterns were then dummy coded into ‘Yes’ (1) and ‘No’ (0) categories based on consistency of responses across the three questions with the rest of the answers treated as missing. A detailed coding process is illustrated in Supporting Information S1: Figure S1.

#### Outcome measures

The survey included three main questions relating to 20 different potential sources of support. Answers to these questions were used to identify each participant's help sought, received, and the perceived helpfulness of help received.

##### Help‐seeking

Participants were asked whether, in the last 12 months, they had sought support for a mental health problem from various sources, including friends and family (7 items), school services (6 items), and NHS, online and/or other services (10 items). This measure was adapted from the Actual Help Seeking Questionnaire (AHSQ; Rickwood et al., 2005; Rickwood & Braithwaite, 1994); to include the UK relevant and online sources of support. Participants were able to select multiple sources of support. After recoding, this resulted in 20 items for available support sources with ‘Yes’ (1), ‘No’ (0), or missing response options for each. These items were then further grouped into formal (e.g., NHS or school services), informal (e.g., friends and family), and online types of support sources (for a full list of items and their recoding see Supporting Information S1: Table S2).

##### Receipt of support

For each selected help‐seeking item (other than the one indicating that no support was sought), participants were asked ‘Regarding this support, are you’: Currently being offered support; Previously been offered support; Not been offered support/been turned away; Changed mind before getting the support. Responses indicating that participants were currently or previously offered support were coded as ‘Offered’ (1), responses indicating that support was not offered or that the participant was turned away were coded as ‘Not offered’ (0) resulting in 20 binary items. Responses of ‘Changed mind before getting the support’ or those indicating that the support was not sought (‘No’ in Seeking help) or unknown (‘Missing’ in Seeking help) were treated as missing.

##### Perceived helpfulness

For each selected help‐seeking item (other than the one indicating that no support was sought), participants were also asked ‘Regarding this support, was it helpful?’. Responses to these 20 items were coded on a 5‐point Likert scale ranging from −2 = ‘Not helpful at all’ to 2 = ‘Very helpful’. Responses to items where the support was not sought (‘No’ in Seeking help), not offered (Receipt of support) or unknown (‘Missing’ in Seeking help or Receipt of support) were again treated as missing.

#### Demographic and descriptor information

Self‐reported information on a range of other variables were used for sample description purposes (see Supporting Information S1: Table S3). This included information on gender, age, ethnicity, country of birth (respondent and their parent[s]), food poverty, and self‐identified mental health problems.

### Data analysis

Data were organised and analysed using R version 4.1.3 (R Development Core Team, 2015). Mixed‐effects models were used to account for clustering within participants across multiple sources of support. We conducted three separate mixed‐effect models with seeking help, presence of the support, and perceived helpfulness as outcome variables via *lme4* package (Bates et al., 2015). Specifically, we used the *glmer* function to conduct mixed‐effect binomial logistic regression on help‐seeking and presence of the support binary data and *lmer* to conduct a linear mixed‐effect model for the perceived helpfulness. All three models consisted of a two‐level structure. Data from the 20 items corresponding to help‐seeking sources were modelled at the first level with the type of support as fixed effects (informal or online with formal support as a reference), which was then nested within students with information on self‐harm and neurodivergence as fixed effects. Confounders such as age, gender, and socioeconomic status were balanced across groups via sensitivity analyses using propensity score matching. Specifically, a proportion of those without self‐harm history were matched to those with self‐harm history (target population) based on year group, gender, ethnicity, country of birth, food poverty, mental health problems, and neurodivergence. One‐to‐one nearest neighbour matching approach via *MatchIt* package has been used to create the matched groups (Ho et al., 2011).

As a proxy for a coefficient of determination (*R* ^2^ in fixed effects models), we report pseudo‐R^2^ coefficients for mixed‐effect models (Nakagawa & Schielzeth, 2013). The marginal *R* ^2^ quantifies outcome variance accounted for by fixed effects (predictor variables), whereas the conditional *R* ^2^ quantifies outcome variance accounted for by the fixed and random effects (i.e., varying intercept between participants) together.

### Ethics approval

The authors assert that all procedures contributing to this work comply with the ethical standards of the relevant national and institutional committees on human experimentation and with the Helsinki Declaration of 1975, as revised in 2013. All procedures involving human subjects/patients were approved by the research ethics committee of the University of Oxford (R62366/RE0014).

## RESULTS

In total, 8506 (69.7%) adolescents in the final sample self‐reported neither history of self‐harm nor neurodivergence. When taken separately, 2129 (17.4%) respondents self‐reported having self‐harmed and 2575 (21.1%) self‐identified as neurodivergent. In combination, a total of 1001 (8.2%) self‐reported both a history of self‐harm and self‐identified as being neurodivergent, corresponding to 47.0% and 38.9% of those reporting self‐harm or self‐identified neurodivergence, respectively. Participant characteristics for the full sample, and according to self‐harm and self‐identified neurodivergence information, are described in Table 1 (see Supporting Information S1: Table S4, for participant characteristics per self‐harm or self‐identified neurodivergence information, separately).

**TABLE 1:** Sample characteristics by self‐harm and self‐identified neurodivergence status combined.

	Total sample (*n* = 12,209)	No self‐harm		Self‐harm	
		Not neurodivergent (*n* = 8506)	Neurodivergent (*n* = 1574)	Not neurodivergent (*n* = 1128)	Neurodivergent (*n* = 1001)
Age *[How old are you?]*					
*M* (SD)	13.9 (1.85)	13.8 (1.82)	14.0 (1.88)	14.4 (1.87)	14.4 (1.87)
Gender *[What is your gender?]*					
Boy	5733 (47.0%)	4251 (50.0%)	955 (60.7%)	268 (23.8%)	259 (25.9%)
Girl	6043 (49.5%)	4115 (48.4%)	546 (34.7%)	815 (72.3%)	567 (56.6%)
Other	196 (1.6%)	32 (0.4%)	29 (1.8%)	24 (2.1%)	111 (11.1%)
Missing	237 (2.0%)	108 (1.3%)	44 (2.8%)	21 (1.9%)	64 (6.4%)
Ethnicity *[What is your ethnic group?]*					
White/White British	6543 (53.6%)	4260 (50.1%)	1025 (65.1%)	606 (53.7%)	717 (65.3%)
Mixed/multiple ethnic groups	688 (5.6%)	434 (5.1%)	96 (6.1%)	76 (6.7%)	82 (8.2%)
Asian/Asian British	1959 (16.0%)	1646 (19.4%)	73 (4.6%)	189 (16.8%)	51 (5.1%)
Black/Black British/African/Caribbean	570 (4.7%)	468 (5.5%)	27 (1.7%)	54 (4.8%)	21 (2.1%)
Arab/Other ethnic group	471 (3.9%)	368 (4.3%)	47 (3.0%)	32 (2.8%)	24 (2.4%)
Missing	1978 (16.2%)	1330 (15.6%)	306 (19.5%)	171 (15.2%)	171 (17.1%)
Born in UK *[Were you born in the UK?]*					
No	1889 (15.5%)	1505 (17.7%)	117 (7.4%)	173 (15.3%)	94 (9.4%)
Yes	10,133 (83.0%)	6883 (80.9%)	1426 (90.6%)	939 (83.2%)	885 (88.4%)
Missing	187 (1.5%)	118 (1.4%)	31 (2.0%)	16 (1.4%)	22 (2.2%)
Parents born in UK *[Were your parents born in the UK?]*					
Neither parent	3606 (29.5%)	2939 (34.6%)	193 (12.3%)	329 (29.2%)	145 (14.5%)
Yes, both parents	6550 (53.6%)	4190 (49.3%)	1111 (70.6%)	599 (53.1%)	650 (64.9%)
Yes, one parent	1755 (14.4%)	1189 (14.0%)	220 (14.0%)	179 (15.9%)	167 (16.7%)
Missing	298 (2.4%)	188 (2.2%)	50 (3.2%)	21 (1.9%)	39 (3.9%)
Food poverty *[At home, I go to bed hungry because there is not enough food in the house.]*					
No	11,660 (95.5%)	8278 (97.3%)	1496 (95.0%)	1026 (91.0%)	860 (85.9%)
Yes	409 (3.3%)	142 (1.7%)	52 (3.3%)	88 (7.8%)	127 (12.7%)
Missing	140 (1.1%)	86 (1.0%)	26 (1.7%)	14 (1.2%)	14 (1.4%)
Self‐identified mental health problems *[Do you think you've had a mental health problem that has affected your daily life?]*					
No	7855 (64.3%)	6635 (78.0%)	869 (55.2%)	261 (23.1%)	90 (9.0%)
Yes	3678 (30.1%)	1445 (17.0%)	588 (37.4%)	794 (70.4%)	851 (85.0%)
Missing	676 (5.5%)	426 (5.0%)	117 (7.4%)	73 (6.5%)	60 (6.0%)
Special educational needs *[Do you receive support for special education needs (e.g. have an EHCP: Education, Health and Care Plan)?]*					
No	10,253 (84.0%)	7819 (91.9%)	826 (52.5%)	1040 (92.2%)	568 (56.7%)
Yes	1002 (8.2%)	282 (3.3%)	447 (28.4%)	38 (3.4%)	235 (23.5%)
Missing	954 (7.8%)	405 (4.8%)	301 (19.1%)	50 (4.4%)	198 (19.8%)
Help‐seeking					
*M* (SD)	0.68 (1.40)	0.38 (0.95)	0.73 (1.41)	1.52 (1.93)	2.22 (2.23)
Total sought	3469 (28.4%)	1623 (19.1%)	504 (32.0%)	635 (56.3%)	707 (70.6%)
Formal	1449 (11.9%)	437 (5.1%)	200 (12.7%)	341 (30.2%)	471 (47.1%)
Informal	3080 (25.2%)	1479 (17.4%)	453 (28.8%)	542 (48.0%)	606 (60.5%)
Online	627 (5.1%)	168 (2.0%)	71 (4.5%)	176 (15.6%)	212 (21.2%)
Receipt of support [sub‐sample]	[*n* = 3062]	[*n* = 1394]	[*n* = 437]	[*n* = 587]	[*n* = 644]
Total offered	2864 (93.5%)	1334 (95.7%)	405 (92.7%)	533 (90.8%)	592 (91.9%)
Formal	1163 (38.0%)	349 (25.0%)	161 (36.8%)	272 (46.3%)	381 (59.2%)
Informal	2510 (82.0%)	1220 (87.5%)	364 (83.3%)	434 (73.9%)	492 (76.4%)
Online	468 (15.3%)	113 (8.1%)	53 (12.1%)	136 (23.2%)	166 (25.8%)
Perceived helpfulness [sub‐sample]	[*n* = 2840]	[*n* = 1323]	[*n* = 399]	[*n* = 529]	[*n* = 589]
Total: *M* (SD)	0.68 (1.01)	1.02 (0.86)	0.76 (0.93)	0.33 (1.05)	0.18 (1.02)
Formal					
*M* (SD)	0.21 (1.21)	0.69 (1.14)	0.44 (1.22)	> 0.01 (1.17)	−0.19 (1.12)
Missing	1693 (59.6%)	979 (74.0%)	239 (59.9%)	260 (49.1%)	215 (36.5%)
Informal					
*M* (SD)	0.79 (0.98)	1.06 (0.84)	0.82 (0.89)	0.47 (1.06)	0.37 (1.05)
Missing	354 (12.5%)	113 (8.5%)	42 (10.5%)	98 (18.5%)	101 (17.1%)
Online					
*M* (SD)	0.48 (1.20)	0.93 (1.01)	0.83 (1.23)	0.32 (1.20)	0.19 (1.19)
Missing	2379 (83.8%)	1212 (91.6%)	346 (86.7%)	395 (74.7%)	426 (72.3%)

*Note*: ‘Missing’ category is comprised of students who declined to respond or those who endorsed other categories, such as ‘Prefer not to say’, or ‘Not sure’.

**FIGURE 1:**
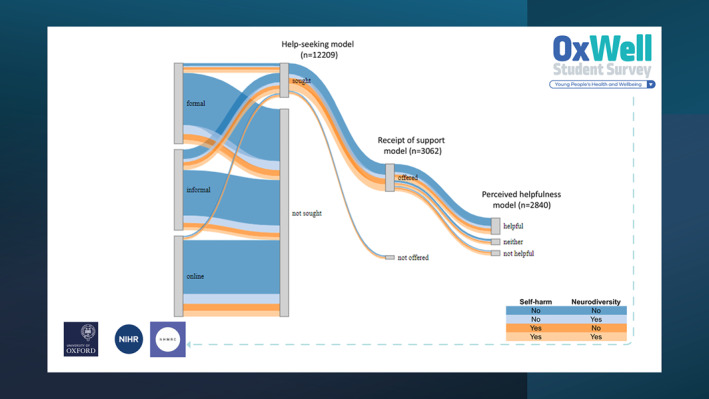
Summary Sankey chart showing help‐seeking, receipt of support, and perceived helpfulness among young people, with differences illustrated by type of support and by self‐harm and self‐identified neurodivergence status.

More than a quarter of participants (28.4%) in the overall sample reported seeking help from at least one of the sources listed (Figure 1). Across groups this ranged from 19.1% of those reporting neither self‐identified neurodivergence nor self‐harm to 70.6% of those reporting both self‐identified neurodivergence and self‐harm (Table 1). Whilst the number of sources where help was sought ranged from 0 to 18 out of a possible 20, the average number of sources per participant ranged from 0.38 in those reporting neither self‐identified neurodivergence (non‐ND) nor self‐harm to 2.22 in those reporting both self‐identified neurodivergence (ND) and self‐harm. In general, the most commonly sought sources of support in each group were parents, stepparents, or carers (11.8%–36.8% per group) and in‐person friends (8.2%–33.4%) (for a full breakdown see Supporting Information S1: Table S5). Participants in all four groups were also relatively likely to help‐seek from pastoral staff at school (2.6%–24.2%). Otherwise, those with no history of self‐harm reported turning to their siblings (non‐ND: 3.8%; ND: 4.6%) or other adults in school (non‐ND: 2.1%; ND: 5.1%). Those reporting self‐harm, however, more commonly turned to CAMHS (non‐ND: 10.6%; ND: 25.3%), their family doctor (GP) (non‐ND: 10.4%; ND: 13.2%), or a private counsellor/therapist (non‐ND: 9.0%; ND: 13.4%).

### Help‐seeking

**FIGURE 2:**
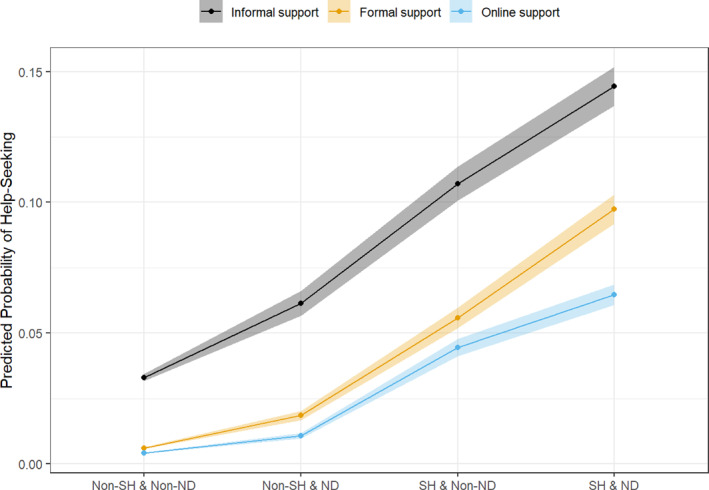
Model predicted probabilities of help‐seeking according to self‐harm (SH) and self‐identified neurodivergence (ND) status by support type.

Both self‐harm (*B* = 2.67, SE = 0.09, OR = 14.37, *p* < 0.001) and self‐identified neurodivergence (*B* = 1.18, SE = 0.10, OR = 3.26, *p* < 0.001) significantly predicted a higher likelihood of help‐seeking. On average, participants were significantly more likely to seek informal than formal help (*B* = 1.88, SE = 0.05, OR = 6.55, *p* < 0.001) and less likely to seek online than formal help (*B* = −0.38, SE = 0.09, OR = 0.68, *p* < 0.001). Yet, this inclination towards informal help‐seeking was significantly reduced in those who reported self‐harm (*B* = −1.09, SE = 0.08, OR = 0.34, *p* < 0.001) and those who self‐identified as neurodivergent (*B* = −0.48, SE = 0.09, OR = 0.62, *p* < 0.001). There was also a significant interaction between self‐harm and self‐identified neurodivergence (*B* = −0.38, SE = 0.09, OR = 0.68, *p* = 0.007). Specifically, the effect of self‐identified neurodivergence on help‐seeking was more pronounced in those who reported self‐harm than those who did not (Figure 2). None of the other interaction effects in the model were significant (see Supporting Information S1: Table S6). Overall, the fixed effects (self‐harm, self‐identified neurodivergence, support type, and their interactions) accounted for 21% of the variance (Nakagawa marginal *R* ^2^ = 0.21) in help‐seeking behaviours. The full model with combined fixed and random effects (i.e., varying intercept between participants), instead, accounted for 60% of the variance (Nakagawa conditional *R* ^2^ = 0.60).

### Receipt of support

**FIGURE 3:**
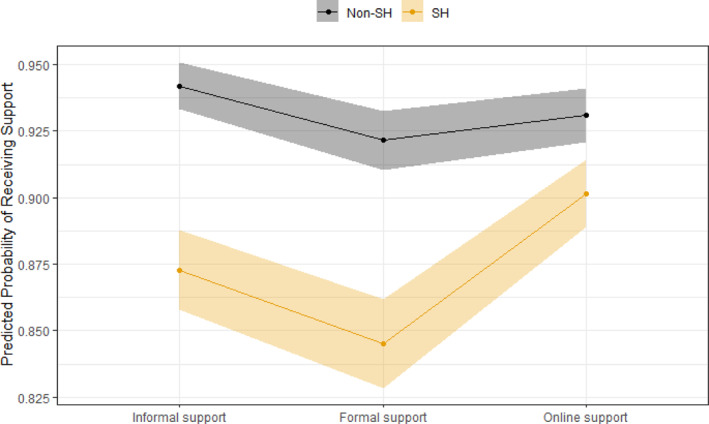
Model predicted probabilities of support offered according to self‐harm (SH) status by support type.

The receipt of support analysis was carried out on the sub‐sample of participants (*n* = 3062) who indicated having sought help from at least one of the 20 sources in the past 12 months. The likelihood of being offered the support was higher for informal than formal sources (*B* = 1.39, SE = 0.37, OR = 4.01, *p* < 0.001). There was also a significant interaction between self‐harm and receiving online help (*B* = 1.98, SE = 0.87, OR = 7.26, *p* = 0.023). Those reporting self‐harm were more likely than those who did not report self‐harm to be offered support from online than formal sources (Figure 3). None of the other main or interaction effects in the model were significant (see Supporting Information S1: Table S6). The fixed effects accounted for only 1% (Nakagawa marginal *R* ^2^ = 0.01), while combined fixed and random effects accounted for 96% (Nakagawa conditional *R* ^2^ = 0.96) of the variance in the receipt of support model.

### Perceived helpfulness

**FIGURE 4:**
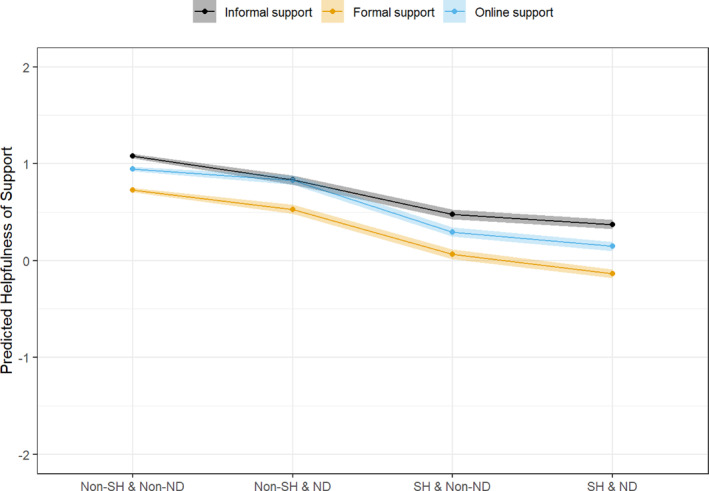
Model predicted perceived helpfulness according to self‐harm (SH) and neurodivergence (ND) status by support type.

The perceived helpfulness analysis was carried out on a further sub‐sample of participants (*n* = 2840) who indicated having sought *and been offered* help from at least one of the 20 sources in the last 12 months. Self‐harm (*B* = −0.66, SE = 0.07, *p* < 0.001) and self‐identified neurodivergence (*B* = −0.20, SE = 0.09, *p* = 0.024) both significantly predicted lower perceived helpfulness of the received support. Both, informal (*B* = 0.35, SE = 0.05, *p* < 0.001) and online (*B* = 0.22, SE = 0.10, *p* = 0.032) support, were perceived as more helpful than the support from the formal sources (Figure 4). None of the interaction effects in the model, however, were significant (see Supporting Information S1: Table S6). The fixed effects accounted for 13% (Nakagawa marginal *R* ^2^ = 0.13) of the perceived helpfulness variance, while combined fixed and random effects accounted for 48% (Nakagawa conditional *R* ^2^ = 0.48) of its variance.

### Sensitivity analyses

Two different sensitivity analyses were conducted to supplement the main findings. First analysis was conducted on a sub‐sample of adolescents who self‐identified as having mental health problems (SA1: *n* = 3678). The second sensitivity analysis was conducted on a sub‐sample balanced for personal characteristics via propensity score matching (SA2: *n* = 4258). The sub‐sample characteristics for both sensitivity analyses are displayed in Table S7.

Both sensitivity analyses resulted in findings that were largely similar to the main analysis (see Supporting Information S1: Tables S8 and S9 for full model estimates). Similarly to the main analyses, help‐seeking was more pronounced in self‐identified neurodivergent adolescents (SA1: *B* = 0.39, SE = 0.10, OR = 1.48, *p* < 0.001; SA2: *B* = 0.33, SE = 0.11, OR = 1.40, *p* = 0.002) and those who self‐harm (SA1: *B* = 1.05, SE = 0.06, OR = 2.87, *p* < 0.001; SA2: *B* = 0.90, SE = 0.09, OR = 2.47, *p* < 0.001). Yet, the interaction between self‐harm and neurodivergence was only significant in the matched (SA2: *B* = 0.35, SE = 0.13, OR = 1.42, *p* = 0.009) rather than mental health problem sub‐sample, suggesting that this finding in the main analysis may be, at least, partially explained by the uneven presence of mental health difficulties in the different groups. Generally, adolescents were again most likely to seek support from informal (SA1: *B* = 1.56, SE = 0.06, OR = 4.78, *p* < 0.001; SA2: *B* = 1.53, SE = 0.07, OR = 4.61, *p* < 0.001) than formal sources and least likely to seek help online (SA1: *B* = −0.65, SE = 0.12, OR = 0.52, *p* < 0.001; SA2: *B* = −0.65, SE = 0.13, OR = 0.52, *p* < 0.001). The inclination towards informal help was again less pronounced in those who reported either self‐harm (SA1: *B* = −0.92, SE = 0.09, OR = 0.40, *p* < 0.001; SA2: *B* = −0.77, SE = 0.09, OR = 0.46, *p* < 0.001) or self‐identified neurodivergence (SA1: *B* = −0.33, SE = 0.11, OR = 0.72, *p* = 0.002; SA2: *B* = −0.27, SE = 0.11, OR = 0.76, *p* = 0.011), in line with main analysis. Differently from the main analysis, however, both sensitivity analyses also yielded a significant interaction effect between self‐harm and online support (SA1: *B* = 0.34, SE = 0.15, OR = 1.40, *p* = 0.024; SA2: *B* = 0.40, SE = 0.15, OR = 1.49, *p* = 0.009). Suggesting that adolescents who self‐harm may be more likely to seek support online than those who do not self‐harm when controlling for the presence of mental health problems. Consistently with the main findings, all adolescents, who sought support from these sources, were more likely to be offered informal than formal support (SA1: *B* = 1.21, SE = 0.42, OR = 3.36, *p* = 0.004; SA2: *B* = 1.43, SE = 0.45, OR = 4.16, *p* = 0.001) and those who reported self‐harm were also more likely to be offered online support than formal support (SA1: *B* = 2.09, SE = 0.99, OR = 8.06, *p* = 0.034; SA2: *B* = 1.99, SE = 0.98, OR = 7.34, *p* = 0.042). Only informal (SA1: *B* = 0.37, SE = 0.06, *p* < 0.001; SA2: *B* = 0.41, SE = 0.07, *p* < 0.001) and not online support, however, was perceived as more helpful than formal support in both sensitivity analyses. Also, only adolescents who self‐harmed (SA1: *B* = −0.57, SE = 0.09, *p* < 0.001; SA2: *B* = −0.50, SE = 0.09, *p* < 0.001) and not necessarily those who self‐identified as neurodivergent reported lower perceived helpfulness than their peers across the different sources of support.

## DISCUSSION

The current study aimed to answer three questions: who seeks help, who receives it, and who finds it helpful? We examined these questions across adolescents who self‐harm, self‐identify as neurodivergent, or both in comparison to their peers, and by type of support (formal, informal, online). By comparing these intersecting identities, we aimed to identify specific disparities and inform service adaptations. Help‐seeking was more pronounced in self‐identified neurodivergent adolescents and those who reported self‐harming and, especially, in those who reported both neurodivergence and self‐harm. All adolescents were more likely to seek support from informal than formal sources and least likely to seek help online. Yet, this inclination towards informal help was less pronounced in those who reported one of either self‐harm or self‐identified neurodivergence. When sought, informal support was offered more often than formal support and, as expected, this did not differ based on adolescent self‐harm or self‐identified neurodivergence status. A pattern of increased receipt of online support was observed for adolescents who self‐harmed but not those who self‐identified as neurodivergent. Both informal and online support were perceived as more helpful than formal support, and this did not differ based on self‐harm or neurodivergence self‐reports. Yet, both adolescents who self‐harmed and those who self‐identified as neurodivergent reported lower perceived helpfulness than their peers across all the different sources of support.

To our knowledge, this is the first study comparing whether and how self‐identified neurodivergent adolescents seek mental health support from informal, formal, and online sources, as well as how this help‐seeking differs based on self‐harm behaviour. Overall, our findings are in line with previous research showing higher overall formal service use (Weiss et al., 2018; Zerbo et al., 2019) and lower satisfaction with services in neurodivergent adults and parents of neurodivergent children (Crane et al., 2019; Jackson et al., 2020; Vogan et al., 2017). Our findings build on this previous literature and demonstrate that this also applies to self‐reports and self‐identification of neurodivergent adolescents. Furthermore, in comparison to their peers, self‐identified neurodivergent adolescents were not only more likely to seek help from formal health and community sources, such as pastoral school staff or mental health services, but also more likely to seek help overall, especially if they had a history of self‐harm. Regardless of the source, however, self‐identified neurodivergent adolescents rated the received support as less helpful relative to their peers. In other words, one third (without self‐harm history) to two thirds (with self‐harm history) of self‐identified neurodivergent adolescents reported turning to someone for mental health support. Thus, it must not be overlooked that many continue to perceive their mental health needs as unmet.

Self‐identified neurodivergent adolescents were more likely to seek mental health support online in comparison to their peers. Yet, similarly to their peers, they were still more likely to seek help from informal or formal than online sources of support. This stands in contrast to some previous research that suggests the internet may be a key mode of potential provision for autistic individuals (Howard & Sedgewick, 2021) and may be particularly beneficial as a mode of support for autistic or ADHD adolescents and young adults (Sehlin et al., 2018). It is possible that our findings are affected by the use of broader neurodivergence self‐identification that included dyslexic and dyspraxic adolescents, who may have a different level of need or perceive written online communication to be less beneficial. Yet, all participants, regardless of their self‐identified neurodivergence, were marginally more likely to be offered support when seeking it online and such support was generally perceived as more helpful than the formal support. This is likely to reflect that the burden of unmet need differs between the instances when the support is sought online and when it is sought from formal sources. In other words, those seeking support from formal sources may be seeking help for more serious issues that cannot be as easily addressed in comparison to those who are seeking support online. In combination with being rarely sought, this suggests that online support could play a complementary role, rather than an alternative, to other support options.

Our findings on help‐seeking in adolescents who self‐harm also extend the previous literature by providing new insights on how their help‐seeking for mental health problems compares to adolescents who do not report a history of self‐harm. Previous studies investigating adolescent help‐seeking for self‐harm in community settings have consistently demonstrated that over a third of adolescents who self‐harm do not seek any help and that, when they do, they tend to turn to informal sources rather than formal sources with relatively few turning for support online (Geulayov et al., 2022; Rowe et al., 2014). It has also been shown that adolescents who self‐harm tend to seek professional mental health support more often than those who do not (Tørmoen et al., 2014). Our findings are consistent with these observations and further suggest that, beyond help‐seeking from more sources in general, adolescents who report self‐harm were, in particular, seeking help from health professionals (e.g., CAMHS and GPs) more often than their peers. However, despite seeking for such support more often relative to their peers, adolescents who self‐harm reported finding formal support the least helpful, resulting in particularly low perceived helpfulness if they were also self‐identifying as neurodivergent. This disparity in formal help‐seeking is likely, in part, to reflect the severity of mental health issues associated with self‐harm and suicidal behaviour (Nock et al., 2013), which may inversely contribute to the perceived helpfulness of that support. However, further investigation, including co‐production with neurodivergent young people, will likely be crucial in understanding how to improve the perceived helpfulness of formal support for neurodivergent adolescents who self‐harm. This is especially important as this group are likely to present in increasing numbers to formal services and it is essential that these touchpoints of care are maximised to enhance longer‐term therapeutic opportunities.

### Limitations

The findings of this study should be interpreted in light of several potential limitations. A careful screening process was undertaken to ensure that any individual responses providing conflicting information across the different service use or free text responses were removed from the study, minimising the inaccuracy of this data. Nevertheless, some inaccuracies may still remain. An important limitation is our reliance on self‐identified neurodivergent status. Self‐identification may not correspond to clinical diagnosis, and we cannot confirm the accuracy of these self‐reports. Thus, neither the rates of self‐harm, nor neurodivergence in this study should be taken to represent prevalence. However, self‐identification is relevant to help‐seeking behaviours and mental health interpretation, which are shaped by personal and social identity. The information on self‐identified neurodivergence was also collected with a single question under a broad neurodivergence umbrella due to shared traits, comorbidities, and overlapping challenges in help‐seeking. Participants were not provided with definitions of the listed conditions. It is possible that adolescents may have varying understanding or misconceptions of terms such as dyspraxia or dyslexia, especially in a self‐report format. Furthermore, this approach may obscure differences in clinical severity or lived experience, and we acknowledge that combining ADHD, autism, and dyslexia/dyspraxia could have introduced heterogeneity into our findings. While subgroup analyses were not feasible under our current design, this issue warrants further study.

While the OxWell Student Survey aims to include a diverse sample of school‐aged children and adolescents, data collection relies on adolescent self‐report from students attending participating schools on the day the survey was conducted. As such, the representativeness of the final sample is limited by non‐participation at both the school and student level. We do not have any information from adolescents not attending school, either just for that day or for prolonged periods of time, or for those who chose not to participate for another reason, or those who chose not to answer these specific questions. As a result, our findings may not fully extend to adolescents with attentional or learning difficulties and disabilities or those with other complex needs who are under‐represented in school‐based surveys. Future research is needed to determine whether current findings extend to adolescents who may not be attending school due to complex needs, including self‐harm, sensory difficulties, learning disability, or mental health issues. The inclusion criteria were purposefully broad to allow for group comparisons, and the analysis included any adolescents who provided the required data. Therefore, a proportion of adolescents (especially in the ‘no self‐harm and no neurodivergence’ group) may not be experiencing any mental health difficulties that would require help‐seeking. However, sensitivity analyses were conducted on a sub‐sample of adolescents who self‐identified as having mental health problems and in a sample balanced for personal characteristics via propensity score matching findings resulting in largely similar findings (Supporting Information S1: Tables S7–S9). Finally, a limited number of help‐seeking behaviours were measured. Whilst 20 different types of support sources were included and within‐participant interdependence accounted for, we were unable to disaggregate those categories further. Thus, we could not capture the dose of mental health support sought or received or distinguish when the support seeking may be driven by parents, schools, or social services (e.g., via referrals), or when support may have been initiated by others (e.g., parents or school staff) but declined by the adolescent. Similarly, if the adolescent sought help from, for example, multiple teachers of varying helpfulness, it is unclear how that was represented in their reported helpfulness.

### Practical implications and future directions

Building on these findings, future research can expand our understanding of help‐seeking by exploring the specific psychological, cultural, and structural factors that shape neurodivergent adolescents' who self‐harm and their decisions and perceptions of seeking mental health support. This could include investigating factors that have been previously shown (see Gulliver et al., 2010; Radez et al., 2021) to affect help‐seeking behaviours in general and self‐harm populations, such as emotional competence (e.g., the ability to recognise and articulate emotions), mental health literacy (e.g., awareness of symptoms and knowledge of when and how to seek help), and previous experiences with support. There is also scope to examine how cultural norms, stigma, and family patterns of service use, alongside practical considerations such as service availability, referral pathways, and perceived accessibility, may differently influence these behaviours in this group of adolescents. A more nuanced understanding of these factors will be essential for designing responsive, equitable support systems that meet the needs of adolescents with complex or intersecting mental health challenges.

The current findings suggest that the disparities in help‐seeking for mental health difficulties in neurodivergent adolescents and those who self‐harm may be occurring at the level of self‐report acceptability of support received rather than their active help‐seeking behaviours or the availability of support when sought. This echoes the priorities of the neurodivergent community themselves calling for more research into understanding how mental health difficulties develop and present in neurodivergent individuals and how services and mental health interventions can be adapted to meet these needs (Cage et al., 2024; James Lind Alliance Priority Setting Partnerships for Autism, 2016; Ostaszewska & Harper, 2024). These findings highlight that both neurodivergent adolescents and those who self‐harm merit special consideration in the design and evaluation of mental health interventions and services. All adolescents were predominately help‐seeking from family and friends, but neurodivergent adolescents and those who self‐harm were also likely to turn to their school community and medical professionals. This emphasises the importance of enabling individuals in young people's immediate interpersonal environment, such as parents, to understand how best to provide appropriate support for mental health difficulties in neurodivergent and non‐neurodivergent adolescents (Creswell et al., 2024). This also highlights a need to further understand the complex interactions between the interpersonal, community, and institutional systems that all play an important role in child and adolescent mental health (Fazel & Soneson, 2023); especially when mental health support is needed for those with intersecting needs, such as self‐harm and neurodivergence.

### Conclusion

This study examined three fundamental questions about adolescent mental health support: who seeks help, who receives it, and who finds it helpful. Our findings demonstrate that self‐identified neurodivergent adolescents and those who self‐harm actively seek support at higher rates than their peers across formal, informal, and online sources, and generally receive it when requested. However, both groups consistently reported lower perceived helpfulness across all support types compared to their peers, with self‐identified neurodivergent adolescents who self‐harm showing the most pronounced disparities despite the highest help‐seeking rates. These results reveal a critical paradox in mental health disparities. While all adolescents were more likely to favour informal support and found it more helpful than formal options, the groups with the greatest need showed lower satisfaction across all support sources but particularly for formal services. The findings point towards the need for more nuanced, individualized approaches that acknowledge the limitations of the available support while building on the relative strengths of informal networks and online resources. Further investigation into how the entire support ecosystem can be adapted to better support the needs of adolescents with complex and intersecting needs to improve their perceptions of helpfulness and ensuring more effective care.

## AUTHOR CONTRIBUTIONS

**Simona Skripkauskaite**: Conceptualization; data curation; formal analysis; investigation; methodology; visualization; writing—original draft; writing—review and editing. **Galit Geulayov**: Conceptualization; investigation; writing—reivew and editing. **Mina Fazel**: Conceptualization; funding acquisition; investigation; project administration; writing—review and editing. **Rohan Borschmann**: Conceptualization; funding acquisition; investigation; resources; writing—review and editing.

## CONFLICT OF INTEREST STATEMENT

The authors declare no conflicts of interest.

## ETHICAL CONSIDERATIONS

Participating schools are primarily recruited directly by local authorities, who enrol participants on the basis of either (1) a parental opt‐out model with student assent (for under 16‐year‐olds); or (2) students' informed consent (for those 16‐years and older). All procedures involving human subjects/patients were approved on January 19, 2023 by the Medical Sciences Interdivisional Research Ethics Committee (MS IDREC) of the University of Oxford (R62366/RE0014).

## Supporting information

Supplementary Material S1

## Data Availability

The data are available from the BrainWaves Hub, following application to and review of an application form. The full list of questions as well as other details are available on a project‐specific OxWell Open Science Framework website (https://osf.io/sekhr/) along with the study protocol.
