# Supplementary material for: Patterns of help‐seeking for mental health problems in 1001 self‐identified neurodivergent adolescents who self‐harm
Source: JCPP Adv. 2025 Oct 7;6(2):e70050. doi: 10.1002/jcv2.70050 (PMC13260676; doi:10.1002/jcv2.70050)
Supplement: Supplementary file 1 — Supplementary Material S1 [file JCV2-6-e70050-s001.docx]

**Table S1.** *Sample attrition characteristics*

|  | Eligible sample ^a^ | Excluded participants | | | | Final total sample |
| --- | --- | --- | --- | --- | --- | --- |
|  |  | Missing self-harm information | Missing neurodiversity information | Missing support seeking information | Contradicting support seeking information ^b^ |  |
|  | (*n*=28702) | (*n*=9116) | (*n*=3953) | (*n*=994) | (*n*=690) | (*n*=12209) |
| Age |  |  |  |  |  |  |
| *M* (*SD*) | 13.8 (1.84) | 13.6 (1.85) | 13.6 (1.76) | 13.7 (1.76) | 13.8 (1.87) | 13.9 (1.85) |
| Gender |  |  |  |  |  |  |
| Boy | 12938 (45.1%) | 4005 (43.9%) | 1650 (41.7%) | 485 (48.8%) | 268 (38.8%) | 5733 (47.0%) |
| Girl | 14238 (49.6%) | 4451 (48.8%) | 2023 (51.2%) | 465 (46.8%) | 382 (55.4%) | 6043 (49.5%) |
| Other | 585 (2.0%) | 213 (2.3%) | 103 (2.6%) | 12 (1.2%) | 30 (4.4%) | 196 (1.6%) |
| Missing | 941 (3.3%) | 447 (4.9%) | 177 (4.5%) | 32 (3.2%) | 10 (1.4%) | 237 (2.0%) |
| Ethnicity |  |  |  |  |  |  |
| White/White British | 15215 (53.0%) | 4639 (50.9%) | 2307 (58.4%) | 504 (50.7%) | 320 (46.4%) | 6543 (53.6%) |
| Mixed/Multiple Ethnic Groups | 1633 (5.7%) | 572 (6.3%) | 217 (5.5%) | 47 (4.7%) | 27 (3.9%) | 688 (5.6%) |
| Asian/Asian British | 4031 (14.0%) | 1188 (13.0%) | 438 (11.1%) | 126 (12.7%) | 113 (16.4%) | 1959 (16.0%) |
| Black/Black British/African/Caribbean | 1400 (4.9%) | 447 (4.9%) | 164 (4.1%) | 69 (6.9%) | 57 (8.3%) | 570 (4.7%) |
| Arab/Other ethnic group | 1186 (4.2%) | 448 (4.9%) | 132 (3.3%) | 41 (4.1%) | 27 (3.9%) | 471 (3.9%) |
| Missing | 5237 (18.2%) | 1822 (20.0%) | 695 (17.6%) | 207 (20.8%) | 146 (21.2%) | 1978 (16.2%) |
| Born in UK |  |  |  |  |  |  |
| No | 4415 (15.4%) | 1486 (16.3%) | 490 (12.4%) | 162 (16.3%) | 128 (18.6%) | 1889 (15.5%) |
| Yes | 23603 (82.2%) | 7314 (80.2%) | 3363 (85.1%) | 806 (81.1%) | 545 (79.0%) | 10133 (83.0%) |
| Missing | 684 (2.4%) | 316 (3.4%) | 100 (2.5%) | 26 (2.6%) | 17 (2.5%) | 187 (1.5%) |
| Parents born in UK |  |  |  |  |  |  |
| Neither parent | 8163 (28.4%) | 2612 (28.7%) | 973 (24.6%) | 267 (26.9%) | 229 (33.2%) | 3606 (29.5%) |
| Yes, both parents | 15385 (53.6%) | 4666 (51.2%) | 2318 (58.6%) | 534 (53.7%) | 334 (48.4%) | 6550 (53.6%) |
| Yes, one parent | 4003 (13.9%) | 1294 (14.2%) | 493 (12.5%) | 139 (14.0%) | 99 (14.4%) | 1755 (14.4%) |
| Missing | 1151 (4.1%) | 544 (6.0%) | 169 (4.3%) | 54 (5.4%) | 28 (4.1%) | 298 (2.4%) |
| Food poverty |  |  |  |  |  |  |
| No | 26219 (91.3%) | 7713 (84.6%) | 3700 (93.6%) | 915 (92.1%) | 614 (89.0%) | 11660 (95.5%) |
| Yes | 1271 (4.4%) | 500 (5.4%) | 194 (4.9%) | 38 (3.8%) | 60 (8.7%) | 409 (3.3%) |
| Missing | 1212 (4.2%) | 903 (10.0%) | 59 (1.5%) | 41 (4.1%) | 16 (2.3%) | 140 (1.1%) |
| Self-identified mental health problems |  |  |  |  |  |  |
| No | 12593 (43.9%) | 1289 (14.1%) | 1704 (43.1%) | 415 (41.8%) | 331 (48.0%) | 7855 (64.3%) |
| Yes | 8437 (29.4%) | 2063 (21.6%) | 1720 (43.5%) | 129 (13.0%) | 305 (44.2%) | 3678 (30.1%) |
| Missing | 7672 (26.7%) | 5764 (63.2%) | 529 (13.4%) | 450 (45.3%) | 54 (7.8%) | 676 (5.5%) |
| Neurodivergence |  |  |  |  |  |  |
| No | 16522 (57.6%) | 4408 (48.4%) | 0 (0%) | 760 (76.5%) | 475 (68.8%) | 9634 (78.9%) |
| Yes | 5601 (19.5%) | 2082 (22.8%) | 0 (0%) | 234 (23.5%) | 215 (31.2%) | 2575 (21.1%) |
| Missing | 6579 (22.9%) | 2626 (28.8%) | 3953 (100.0%) | 0 (0.0%) | 0 (0.0%) | 0 (0.0%) |
| Self-harm |  |  |  |  |  |  |
| No | 15647 (54.5%) | 0 (0.0%) | 2821 (71.4%) | 858 (86.3%) | 492 (71.3%) | 10080 (82.6%) |
| Yes | 3939 (13.7%) | 0 (0.0%) | 1132 (28.6%) | 136 (13.7%) | 198 (28.7%) | 2129 (17.4%) |
| Missing | 9116 (31.8%) | 9116 (100.0%) | 0 (0.0%) | 0 (0.0%) | 0 (0.0%) | 0 (0.0%) |

*Note***. ^a^** In total, 42,215 students, from 181 schools and colleges across England, consented to the OxWell survey in 2023. Of this group, 9,250 were removed due to falling outside the secondary- or further education-school range (Year groups 7-13), 3,694 were removed due to spending less than 10 min on the survey as the survey could not be reasonably completed in that time, and further 569 participants were removed due to being over 18 years old or not providing information on their age. This has resulted in a sample of 28,702 eligible participants based on pre-defined inclusion criteria (https://osf.io/yckpb). ^b^ Participants who provided conflicting responses, such as identifying that they sought support from multiple sources and that they did not seek any support from any sources, were excluded from the final sample. ‘Missing’ category can include lack of response and/or responses that do not fit in the other categories, such as ‘Prefer not to say’ or ‘Not sure’.

**Fig S1.** *A flowchart describing ascertainment of the final self-harm history variable.*

*
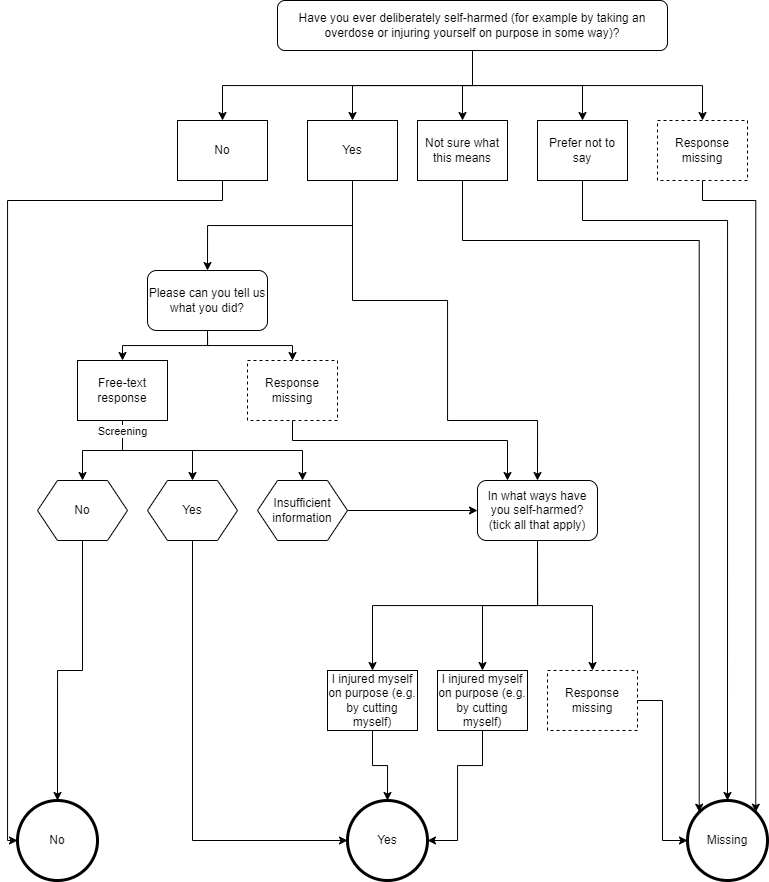
*

*Note.* The free-text responses were screened for validity by study authors (GG and MF) and an independent evaluator (RN). For example, answers indicating accidental damage (e.g., ‘I slipped and fell’) were recoded as not self-harm, whereas those indicating an emotional response rather than method (e.g. ‘I was sad’) were treated as missing.

**Table S2.** *Support seeking measures and their associated coding.*

|  | Items | Recoded responses^a^ | Support type |
| --- | --- | --- | --- |
| *“In the last 12 months, have you tried to ask for support for a mental health problem from the following friends or family (tick all that apply)”* | | | |
|  | Parent, step-parent or carer | Yes \| No \| Missing | Informal |
|  | Sibling(s) | Yes \| No \| Missing | Informal |
|  | Someone else in your family | Yes \| No \| Missing | Informal |
|  | Friend(s), mainly known in person | Yes \| No \| Missing | Informal |
|  | Friend(s), mainly known online | Yes \| No \| Missing | Online |
|  | An adult outside of school/college (at a sport club, another parent, family friend) | Yes \| No \| Missing | Informal |
|  | No | - | - |
| *“In the last 12 months, have you tried to ask for support for a mental health problem from the following school services: (tick all that apply)”* | | | |
|  | School/college Nurse/Counsellor/other pastoral staff at school/college | Yes \| No \| Missing | Formal |
|  | Educational Mental health practitioner (EMHP) | Yes \| No \| Missing | Formal |
|  | Another adult at school/college | Yes \| No \| Missing | Informal |
|  | A peer mentor at school/college | Yes \| No \| Missing | Informal |
|  | Other school/college services (please specify)^b^ | Yes \| No \| Missing | Formal |
|  | No | *-* | *-* |
| *“In the last 12 months, have you tried to ask for support for a mental health problem from the following NHS, online and/or other services: (tick all that apply)”* | | | |
|  | GP (family doctor) | Yes \| No \| Missing | Formal |
|  | Social worker | Yes \| No \| Missing | Formal |
|  | CAMHS (NHS Child and Adolescent Mental Health Services) | Yes \| No \| Missing | Formal |
|  | Private counsellor/therapist | Yes \| No \| Missing | Formal |
|  | Support service given by a charity | Yes \| No \| Missing | Formal |
|  | A telephone/text helpline | Yes \| No \| Missing | Online |
|  | Website or online forum | Yes \| No \| Missing | Online |
|  | From an anonymous user on an online platform/ chatroom/ forum/ server | Yes \| No \| Missing | Online |
|  | Other services (please specify)^b^ | Yes \| No \| Missing | Formal |
|  | No | *-* | *-* |

^a^ If the responded has not indicated the particular category of support but indicated seeking support elsewhere (within that question) or ticked the ‘No’ response, the item was recoded as ‘No’ indicating that support from that source was not sought

^b^ Free text responses to ‘Other school/college services (please specify)’ and ‘Other services (please specify)’ were evaluated by study authors and assigned to one of the other categories when appropriate. When free text responses were judged to not fit in any of the other categories or were not provided, they were coded as either ‘Other school/college services’ or ‘Other services’.

**Table S3.** *Demographic and descriptor information measures and their response options.*

| Question | | Original responses | Recoded responses |
| --- | --- | --- | --- |
| Age | |  |  |
|  | *How old are you?* | |  |
|  |  | 11-18 | Continuous numerical scale |
|  |  | ‘Over 18’ | Excluded |
| Gender | | |  |
|  | *What is your gender?* | |  |
|  |  | Female | Girl |
|  |  | Male | Boy |
|  |  | Prefer not to say | Missing |
|  |  | Prefer to self-identify [free text box] | Other |
| Ethnicity | | |  |
|  | *What is your ethnic group?* | |  |
|  |  | White - English/Welsh/Scottish/Northern Irish/British | White/White British |
|  |  | White - Irish White - Gypsy or Irish Traveller |  |
|  |  | White – Other |  |
|  |  | Mixed/Multiple ethnic groups - White and Black Caribbean | Mixed/Multiple Ethnic Groups |
|  |  | Mixed/Multiple ethnic groups - White and Black African |  |
|  |  | Mixed/Multiple ethnic groups - White and Asian |  |
|  |  | Mixed/Multiple ethnic groups – Other |  |
|  |  | Asian/Asian British – Indian | Asian/Asian British |
|  |  | Asian/Asian British – Pakistani |  |
|  |  | Asian/Asian British – Bangladeshi |  |
|  |  | Asian/Asian British – Chinese |  |
|  |  | Asian/Asian British – Other |  |
|  |  | Black/African/Caribbean/Black British – African | Black/Black British/African/Caribbean |
|  |  | Black/African/Caribbean/Black British – Caribbean |  |
|  |  | Black/African/Caribbean/Black British – Other |  |
|  |  | Arab | Arab/Other ethnic group |
|  |  | Other ethnic group |  |
| Born in UK | | |  |
|  | *Were you born in the UK?* | |  |
|  |  | No | No |
|  |  | Yes | Yes |
|  |  | Prefer not to say | Missing |
| Parents born in UK | | |  |
|  | *Were your parents born in the UK?* | |  |
|  |  | Yes, one parent | Yes, one parent |
|  |  | Yes, both parents | Yes, both parents |
|  |  | Neither parent | Neither parent |
|  |  | Prefer not to say | Missing |
| Food poverty | | |  |
|  | *At home, I go to bed hungry because there is not enough food in the house.* | | |
|  |  | Never or hardly ever | No |
|  |  | Some of the time | Yes |
|  |  | Often |  |
| Self-identified mental health problems | | |  |
|  | *Do you think you've had a mental health problem that has affected your daily life?* | | |
|  |  | No | No |
|  |  | Yes - in the past 12 months | Yes |
|  |  | Yes - more than a year ago |  |
|  |  | Prefer not to say | Missing |

**Table S4.** *Sample characteristics according to self-harm and neurodiversity information, separately*

|  | Self-harm | |  | Neurodiversity | |
| --- | --- | --- | --- | --- | --- |
|  | No | Yes |  | No | Yes |
|  | (*n*=10080) | (*n*=2129) |  | (*n*=9634) | (*n*=2575) |
| Age |  |  |  |  |  |
| *M* (*SD*) | 13.8 (1.83) | 14.4 (1.87) |  | 13.9 (1.84) | 14.1 (1.89) |
| Gender |  |  |  |  |  |
| Boy | 5206 (51.6%) | 527 (24.8%) |  | 4519 (46.9%) | 1214 (47.1%) |
| Girl | 4661 (46.2%) | 1382 (64.9%) |  | 4930 (51.2%) | 1113 (43.2%) |
| Other | 61 (0.6%) | 135 (6.3%) |  | 56 (0.6%) | 140 (5.4%) |
| Missing | 152 (1.5%) | 85 (4.0%) |  | 129 (1.3%) | 108 (4.2%) |
| Ethnicity |  |  |  |  |  |
| White/White British | 5285 (52.4%) | 1258 (59.1%) |  | 4866 (50.5%) | 1677 (65.1%) |
| Mixed/Multiple Ethnic Groups | 530 (5.3%) | 158 (7.4%) |  | 510 (5.3%) | 178 (6.9%) |
| Asian/Asian British | 1719 (17.1%) | 240 (11.3%) |  | 1835 (19.0%) | 124 (4.8%) |
| Black/Black British/African/Caribbean | 495 (4.9%) | 75 (3.5%) |  | 522 (5.4%) | 48 (1.9%) |
| Arab/Other ethnic group | 415 (4.1%) | 56 (2.6%) |  | 400 (4.2%) | 71 (2.8%) |
| Missing | 1636 (16.2%) | 342 (16.1%) |  | 1501 (15.6%) | 477 (18.5%) |
| Born in UK |  |  |  |  |  |
| No | 1622 (16.1%) | 267 (12.5%) |  | 1678 (17.4%) | 211 (8.2%) |
| Yes | 8309 (82.4%) | 1824 (85.7%) |  | 7822 (81.2%) | 2311 (89.7%) |
| Missing | 149 (1.5%) | 38 (1.8%) |  | 134 (1.4%) | 53 (2.1%) |
| Parents born in UK |  |  |  |  |  |
| Neither parent | 3132 (31.1%) | 474 (22.3%) |  | 3268 (33.9%) | 338 (13.1%) |
| Yes, both parents | 5301 (52.6%) | 1249 (58.7%) |  | 4789 (49.7%) | 1761 (68.4%) |
| Yes, one parent | 1409 (14.0%) | 346 (16.3%) |  | 1368 (14.2%) | 338 (13.1%) |
| Missing | 238 (2.4%) | 60 (2.8%) |  | 209 (2.2%) | 89 (3.5%) |
| Food poverty |  |  |  |  |  |
| No | 9774 (97.0%) | 1886 (88.6%) |  | 9304 (96.6%) | 2356 (91.5%) |
| Yes | 194 (1.9%) | 215 (10.1%) |  | 230 (2.4%) | 179 (7.0%) |
| Missing | 112 (1.1%) | 28 (1.3%) |  | 100 (1.0%) | 40 (1.6%) |
| Self-identified mental health problems |  |  |  |  |  |
| No | 7504 (74.4%) | 351 (16.5%) |  | 6896 (71.6%) | 959 (37.2%) |
| Yes | 2033 (20.2%) | 1645 (77.3%) |  | 2239 (23.2%) | 1439 (55.9%) |
| Missing | 543 (5.4%) | 133 (6.2%) |  | 499 (5.2%) | 177 (6.9%) |

*Note.* ‘Missing’ category can include lack of response and/or responses that do not fit in the other categories, such as ‘Prefer not to say’ or ‘Not sure’.

**Table S5.** *Proportion of participants seeking help* *from the different support sources.*

|  | Total sample | No self-harm | |  | Self-harm | |
| --- | --- | --- | --- | --- | --- | --- |
|  |  | Not neurodivergent | Neurodivergent |  | Not neurodivergent | Neurodivergent |
|  | (n=12209) | (n=8506) | (n=1574) |  | (n=1128) | (n=1001) |
| Parent, step-parent or carer | **1983 (16.2%)** | **1003 (11.8%)** | **304 (19.3%)** |  | **308 (27.3%)** | **368 (36.8%)** |
| Friend(s), mainly known in person | **1525 (12.5%)** | **699 (8.2%)** | **204 (13.0%)** |  | **288 (25.5%)** | **334 (33.4%)** |
| School/college Nurse/Counsellor/other pastoral staff at  school/college | **710 (5.8%)** | **217 (2.6%)** | **75 (4.8%)** |  | **176 (15.6%)** | **242 (24.2%)** |
| Sibling(s) | **550 (4.5%)** | **319 (3.8%)** | **73 (4.6%)** |  | 83 (7.4%) | 75 (7.5%) |
| CAMHS (NHS Child and Adolescent Mental Health Services) | **511 (4.2%)** | 68 (0.8%) | 70 (4.4%) |  | **120 (10.6%)** | **253 (25.3%)** |
| Another adult at school/college | 468 (3.8%) | **176 (2.1%)** | **81 (5.1%)** |  | 93 (8.2%) | 118 (11.8%) |
| GP (family doctor) | 414 (3.4%) | 111 (1.3%) | 54 (3.4%) |  | **117 (10.4%)** | 132 (13.2%) |
| Private counsellor/therapist | 380 (3.1%) | 95 (1.1%) | 49 (3.1%) |  | 102 (9.0%) | **134 (13.4%)** |
| Friend(s), known online | 353 (2.9%) | 105 (1.2%) | 49 (3.1%) |  | 86 (7.6%) | 113 (11.3%) |
| Someone else in your family | 265 (2.2%) | 141 (1.7%) | 43 (2.7%) |  | 41 (3.6%) | 40 (4.0%) |
| An adult outside of school (at a sport club, another parent,  family friend) | 262 (2.1%) | 90 (1.1%) | 43 (2.7%) |  | 52 (4.6%) | 77 (7.7%) |
| A telephone/text helpline | 156 (1.3%) | 14 (0.2%) | 12 (0.8%) |  | 57 (5.1%) | 73 (7.3%) |
| Website or online forum | 149 (1.2%) | 42 (0.5%) | 13 (0.8%) |  | 37 (3.3%) | 57 (5.7%) |
| Educational Mental health practitioner (EMHP) | 138 (1.1%) | 39 (0.5%) | 24 (1.5%) |  | 31 (2.7%) | 44 (4.4%) |
| Social worker | 124 (1.0%) | 22 (0.3%) | 13 (0.8%) |  | 31 (2.7%) | 58 (5.8%) |
| A peer mentor at school/college | 92 (0.8%) | 35 (0.4%) | 18 (1.1%) |  | 19 (1.7%) | 20 (2.0%) |
| From an anonymous user on an online platform/ chatroom/ forum/ server | 91 (0.7%) | 21 (0.2%) | 5 (0.3%) |  | 36 (3.2%) | 29 (2.9%) |
| Support service given by a charity | 71 (0.6%) | 14 (0.2%) | 8 (0.5%) |  | 20 (1.8%) | 29 (2.9%) |
| Other school/college services | 36 (0.3%) | 15 (0.2%) | 5 (0.3%) |  | 5 (0.4%) | 11 (1.1%) |
| Other services | 28 (0.2%) | 10 (0.1%) | 7 (0.4%) |  | 4 (0.4%) | 7 (0.7%) |

*Note.* Five most common sources in each group are marked in **Bold**.

**Table S6.** *Summary statistics for mixed-effect binomial logistic regression and linear mixed-effect models.*

|  |  | Help-seeking | | | |  | | Receipt of support | | | | |  | | Perceived helpfulness | | | |  |
| --- | --- | --- | --- | --- | --- | --- | --- | --- | --- | --- | --- | --- | --- | --- | --- | --- | --- | --- | --- |
|  |  | *B* | *SE* | *OR* | *p* | |  | | *B* | *SE* | *OR* | *p* | |  | | *B* | *SE* | *p* | |
| Fixed effects | |  |  |  |  | |  | |  |  |  |  | |  | |  |  |  | |
|  | Self-harm | **2.67** | **0.09** | **14.37** | **<.001** | |  | | -0.68 | 0.46 | 0.51 | .140 | |  | | **-0.66** | **0.07** | **<.001** | |
|  | Neurodivergence | **1.18** | **0.10** | **3.26** | **<.001** | |  | | -0.22 | 0.56 | 0.80 | .697 | |  | | **-0.20** | **0.09** | **.024** | |
|  | Informal support | **1.88** | **0.05** | **6.55** | **<.001** | |  | | **1.39** | **0.37** | **4.01** | **<.001** | |  | | **0.35** | **0.05** | **<.001** | |
|  | Online support | **-0.38** | **0.09** | **0.68** | **<.001** | |  | | 0.01 | 0.67 | 1.00 | .999 | |  | | **0.22** | **0.10** | **.032** | |
|  | Self-harm * Neurodivergence | **-0.38** | **0.14** | **0.68** | **.007** | |  | | 0.39 | 0.70 | 1.47 | .582 | |  | | -<0.01 | 0.11 | .983 | |
|  | Self-harm * Informal support | **-1.09** | **0.08** | **0.34** | **<.001** | |  | | -0.34 | 0.47 | 0.71 | .473 | |  | | 0.06 | 0.08 | .444 | |
|  | Self-harm * Online support | 0.12 | 0.12 | 1.13 | .317 | |  | | **1.98** | **0.87** | **7.26** | **.023** | |  | | 0.01 | 0.13 | .928 | |
|  | Neurodivergence * Informal support | **-0.48** | **0.09** | **0.62** | **<.001** | |  | | -0.45 | 0.58 | 0.64 | .439 | |  | | -0.05 | 0.09 | .585 | |
|  | Neurodivergence * Online support | -0.20 | 0.16 | 0.81 | .197 | |  | | 1.66 | 1.21 | 5.28 | .170 | |  | | 0.08 | 0.17 | .633 | |
|  | Self-harm * Neurodivergence * Informal support | 0.19 | 0.12 | 1.21 | .112 | |  | | -0.14 | 0.69 | 0.87 | .839 | |  | | 0.15 | 0.12 | .209 | |
|  | Self-harm * Neurodivergence * Online support | -0.02 | 0.20 | 0.98 | .924 | |  | | -2.55 | 1.39 | 0.08 | .068 | |  | | -0.03 | 0.21 | .887 | |
| Model Fit | |  |  |  |  | |  | |  |  |  |  | |  | |  |  |  | |
|  | Marginal R^2^ | .21 |  |  |  | |  | | .01 |  |  |  | |  | | .13 |  |  | |
|  | Conditional R^2^ | .60 |  |  |  | |  | | .96 |  |  |  | |  | | .48 |  |  | |
| *n* | |  |  |  |  | |  | |  |  |  |  | |  | |  |  |  | |
|  | Participants | 12209 |  |  |  | |  | | 3062 |  |  |  | |  | | 2840 |  |  | |
|  | Observations | 244180 |  |  |  | |  | | 7148 |  |  |  | |  | | 6333 |  |  | |

*Note.* Formal support was used as a refence category for both Informal and Online support effects.

**Sensitivity Analyses**

**Table S7.** *Sensitivity analyses sub-sample characteristics by self-harm and neurodivergence status combined*

|  |  | Sensitivity Analysis 1 (SA1) | | | | | | | | |  | | Sensitivity Analysis 2 (SA2) | | | | | | | | | |
| --- | --- | --- | --- | --- | --- | --- | --- | --- | --- | --- | --- | --- | --- | --- | --- | --- | --- | --- | --- | --- | --- | --- |
|  |  | No self-harm | | |  | | Self-harm | | | |  | | No self-harm | | | |  | | Self-harm | | | |
|  |  | Not neurodivergent | Neurodivergent | |  | | Not neurodivergent | | Neurodivergent | |  | | Not neurodivergent | | Neurodivergent | |  | | Not neurodivergent | | Neurodivergent | |
|  |  | (n=1445) | (n=588) | |  | | (n=794) | | (n=851) | |  | | (n=1389) | | (n=740) | |  | | (n=1128) | | (n=1001) | |
| Age | |  |  | |  | |  | |  | |  | |  | |  | |  | |  | |  | |
|  | *M* (*SD*) | 14.5 (1.84) | 14.3 (1.87) | |  | | 14.6 (1.86) | | 14.5 (1.85) | |  | | 14.5 (1.84) | | 14.2 (1.88) | |  | | 14.4 (1.87) | | 14.4 (1.87) | |
| Gender | |  |  | |  | |  | |  | |  | |  | |  | |  | |  | |  | |
|  | Boy | 513 (35.5%) | 308 (52.4%) | |  | | 153 (19.3%) | | 198 (23.3%) | |  | | 270 (19.4%) | | 365 (49.3%) | |  | | 268 (23.8%) | | 259 (25.9%) | |
|  | Girl | 907 (62.8%) | 240 (40.8%) | |  | | 612 (77.1%) | | 506 (59.5%) | |  | | 1084 (78.0%) | | 312 (42.2%) | |  | | 815 (72.3%) | | 567 (56.6%) | |
|  | Other | 6 (0.4%) | 18 (3.1%) | |  | | 18 (2.3%) | | 97 (11.4%) | |  | | 24 (1.7%) | | 34 (4.6%) | |  | | 21 (1.9%) | | 64 (6.4%) | |
|  | Missing | 19 (1.3%) | 22 (3.7%) | |  | | 11 (1.4%) | | 50 (5.9%) | |  | | 11 (0.8%) | | 29 (3.9%) | |  | | 24 (2.1%) | | 111 (11.1%) | |
| Ethnicity | |  |  | |  | |  | |  | |  | |  | |  | |  | |  | |  | |
|  | White/White British | 811 (56.1%) | 383 (65.1%) | |  | | 450 (56.7%) | | 562 (66.0%) | |  | | 748 (53.9%) | | 481 (65.0%) | |  | | 606 (53.7%) | | 652 (65.1%) | |
|  | Mixed/Multiple Ethnic Groups | 84 (5.8%) | 35 (6.0%) | |  | | 54 (6.8%) | | 72 (8.5%) | |  | | 86 (6.2%) | | 43 (5.8%) | |  | | 76 (6.7%) | | 82 (8.2%) | |
|  | Asian/Asian British | 234 (16.2%) | 31 (5.3%) | |  | | 115 (14.5%) | | 44 (5.2%) | |  | | 249 (17.9%) | | 36 (4.9%) | |  | | 189 (16.8%) | | 51 (5.1%) | |
|  | Black/Black  British/African/Caribbean | 81 (5.6%) | 11 (1.9%) | |  | | 36 (4.5%) | | 17 (2.0%) | |  | | 73 (5.3%) | | 12 (1.6%) | |  | | 54 (4.8%) | | 21 (2.1%) | |
|  | Arab/Other ethnic group | 45 (3.1%) | 18 (3.1%) | |  | | 24 (3.0%) | | 18 (2.1%) | |  | | 37 (2.7%) | | 21 (2.8%) | |  | | 32 (2.8%) | | 24 (2.4%) | |
|  | Missing | 190 (13.1%) | 110 (18.7%) | |  | | 115 (14.5%) | | 138 (16.2%) | |  | | 196 (14.1%) | | 147 (19.9%) | |  | | 171 (15.2%) | | 171 (17.1%) | |
| Born in UK | |  |  | |  | |  | |  | |  | |  | |  | |  | |  | |  | |
|  | No | 225 (15.6%) | 53 (9.0%) | |  | | 115 (14.5%) | | 79 (9.3%) | |  | | 214 (15.4%) | | 69 (9.3%) | |  | | 173 (15.3%) | | 94 (9.4%) | |
|  | Yes | 1209 (83.7%) | 525 (89.3%) | |  | | 672 (84.6%) | | 756 (88.8%) | |  | | 1162 (83.7%) | | 656 (88.6%) | |  | | 939 (83.2%) | | 885 (88.4%) | |
|  | Missing | 11 (0.8%) | 10 (1.7%) | |  | | 7 (0.9%) | | 16 (1.9%) | |  | | 13 (0.9%) | | 15 (2.0%) | |  | | 16 (1.4%) | | 22 (2.2%) | |
| Parents born in UK | |  |  | |  | |  | |  | |  | |  | |  | |  | |  | |  | |
|  | Neither parent | 430 (29.8%) | 85 (14.5%) | |  | | 217 (27.3%) | | 125 (14.7%) | |  | | 441 (31.7%) | | 105 (14.2%) | |  | | 329 (29.2%) | | 145 (14.5%) | |
|  | Yes, both parents | 801 (55.4%) | 401 (68.2%) | |  | | 437 (55.0%) | | 561 (65.9%) | |  | | 733 (52.8%) | | 512 (69.2%) | |  | | 599 (53.1%) | | 650 (64.9%) | |
|  | Yes, one parent | 201 (13.9%) | 89 (15.1%) | |  | | 130 (16.4%) | | 134 (15.7%) | |  | | 193 (13.9%) | | 103 (13.9%) | |  | | 179 (15.9%) | | 167 (16.7%) | |
|  | Missing | 13 (0.9%) | 13 (2.2%) | |  | | 10 (1.3%) | | 31 (3.6%) | |  | | 22 (1.6%) | | 20 (2.7%) | |  | | 21 (1.9%) | | 39 (3.9%) | |
| Food poverty | |  |  | |  | |  | |  | |  | |  | |  | |  | |  | |  | |
|  | No | 1391 (96.3%) | 557 (94.7%) | |  | | 712 (89.7%) | | 729 (85.7%) | |  | | 1324 (95.3%) | | 692 (93.5%) | |  | | 1026 (91.0%) | | 860 (85.9%) | |
|  | Yes | 43 (3.0%) | 25 (4.3%) | |  | | 76 (9.6%) | | 111 (13.0%) | |  | | 54 (3.9%) | | 38 (5.1%) | |  | | 88 (7.8%) | | 127 (12.7%) | |
|  | Missing | 11 (0.8%) | 6 (1.0%) | |  | | 6 (0.8%) | | 11 (1.3%) | |  | | 11 (0.8%) | | 10 (1.4%) | |  | | 14 (1.2%) | | 14 (1.4%) | |
| Self-identified mental health problems | | | |  | |  | |  | |  | |  | |  | |  | |  | |  | |  |
|  | No | 0 (0%) | 0 (0%) | |  | | 0 (0%) | | 0 (0%) | |  | | 261 (18.8%) | | 86 (11.6%) | |  | | 261 (23.1%) | | 90 (9.0%) | |
|  | Yes | 1445 (100%) | 588 (100%) | |  | | 794 (100%) | | 851 (100%) | |  | | 1055 (76.0%) | | 586 (79.2%) | |  | | 794 (70.4%) | | 851 (85.0%) | |
|  | Missing | 0 (0%) | 0 (0%) | |  | | 0 (0%) | | 0 (0%) | |  | | 73 (5.3%) | | 68 (9.2%) | |  | | 73 (6.5%) | | 60 (6.0%) | |

*Note.* ‘Missing’ category is comprised of students who declined to respond or those who endorsed other categories, such as ‘Prefer not to say’, or ‘Not sure’.

**Table S8.** *Summary statistics for mixed-effect binomial logistic regression and linear mixed-effect models in sensitivity analysis 1 (SA1).*

|  |  | Help-seeking | | | |  | | Receipt of support | | | | |  | | Perceived helpfulness | | | |  |
| --- | --- | --- | --- | --- | --- | --- | --- | --- | --- | --- | --- | --- | --- | --- | --- | --- | --- | --- | --- |
|  |  | *B* | *SE* | *OR* | *p* | |  | | *B* | *SE* | *OR* | *p* | |  | | *B* | *SE* | *p* | |
| Fixed effects | |  |  |  |  | |  | |  |  |  |  | |  | |  |  |  | |
|  | Self-harm | **1.05** | **0.06** | **2.87** | **<.001** | |  | | -0.92 | 0.50 | 0.40 | .066 | |  | | **-0.57** | **0.09** | **<.001** | |
|  | Neurodivergence | **0.39** | **0.10** | **1.48** | **<.001** | |  | | -0.47 | 0.61 | 0.62 | .443 | |  | | *-0.10* | *0.11* | *.330* | |
|  | Informal support | **1.56** | **0.06** | **4.78** | **<.001** | |  | | **1.21** | **0.42** | **3.36** | **.004** | |  | | **0.37** | **0.06** | **<.001** | |
|  | Online support | **-0.65** | **0.12** | **0.52** | **<.001** | |  | | 0.03 | 0.80 | 1.03 | .972 | |  | | *0.11* | *0.14* | *.413* | |
|  | Self-harm * Neurodivergence | *0.13* | *0.13* | *1.14* | *.308* | |  | | 0.64 | 0.74 | 1.90 | .385 | |  | | -0.10 | 0.13 | .445 | |
|  | Self-harm * Informal support | **-0.92** | **0.09** | **0.40** | **<.001** | |  | | -0.11 | 0.52 | 0.89 | .831 | |  | | -0.01 | 0.09 | .938 | |
|  | Self-harm * Online support | ***0.34*** | ***0.15*** | ***1.40*** | ***.024*** | |  | | **2.09** | **0.99** | **8.06** | **.034** | |  | | 0.08 | 0.17 | .648 | |
|  | Neurodivergence * Informal support | **-0.33** | **0.11** | **0.72** | **.002** | |  | | -0.31 | 0.63 | 0.73 | .624 | |  | | -0.06 | 0.11 | .564 | |
|  | Neurodivergence * Online support | -0.12 | 0.20 | 0.89 | .558 | |  | | 0.74 | 1.27 | 2.09 | .560 | |  | | 0.19 | 0.22 | .396 | |
|  | Self-harm * Neurodivergence * Informal support | 0.12 | 0.13 | 1.12 | .382 | |  | | -0.36 | 0.74 | 0.70 | .624 | |  | | 0.20 | 0.14 | .133 | |
|  | Self-harm * Neurodivergence * Online support | -0.05 | 0.23 | 0.95 | .814 | |  | | -1.81 | 1.45 | 0.16 | .211 | |  | | -0.08 | 0.26 | .743 | |
| Model Fit | |  |  |  |  | |  | |  |  |  |  | |  | |  |  |  | |
|  | Marginal R^2^ | .14 |  |  |  | |  | | .01 |  |  |  | |  | | .11 |  |  | |
|  | Conditional R^2^ | .35 |  |  |  | |  | | .94 |  |  |  | |  | | .46 |  |  | |
| *n* | |  |  |  |  | |  | |  |  |  |  | |  | |  |  |  | |
|  | Participants | 3678 |  |  |  | |  | | 2010 |  |  |  | |  | | 1862 |  |  | |
|  | Observations | 73560 |  |  |  | |  | | 5286 |  |  |  | |  | | 4622 |  |  | |

*Note.* Formal support was used as a refence category for both Informal and Online support effects. Lines in **‘Bold’**, indicate significant effects. Lines in *‘Italics’*, indicates the effects that changed significance in comparison to the main analysis.

**Table S9.** *Summary statistics for mixed-effect binomial logistic regression and linear mixed-effect models in sensitivity analysis 2 (SA2).*

|  |  | Help-seeking | | | |  | | Receipt of support | | | | |  | | Perceived helpfulness | | | |  |
| --- | --- | --- | --- | --- | --- | --- | --- | --- | --- | --- | --- | --- | --- | --- | --- | --- | --- | --- | --- |
|  |  | *B* | *SE* | *OR* | *p* | |  | | *B* | *SE* | *OR* | *p* | |  | | *B* | *SE* | *p* | |
| Fixed effects | |  |  |  |  | |  | |  |  |  |  | |  | |  |  |  | |
|  | Self-harm | **0.90** | **0.09** | **2.47** | **<.001** | |  | | -0.66 | 0.51 | 0.52 | .196 | |  | | **-0.50** | **0.09** | **<.001** | |
|  | Neurodivergence | **0.33** | **0.11** | **1.40** | **.002** | |  | | -0.27 | 0.62 | 0.76 | .664 | |  | | *-0.12* | *0.11* | *.281* | |
|  | Informal support | **1.53** | **0.07** | **4.61** | **<.001** | |  | | **1.43** | **0.45** | **4.16** | **.001** | |  | | **0.41** | **0.07** | **<.001** | |
|  | Online support | **-0.65** | **0.13** | **0.52** | **<.001** | |  | | -0.11 | 0.82 | 0.89 | .889 | |  | | *0.18* | *0.15* | *.224* | |
|  | Self-harm * Neurodivergence | **0.35** | **0.13** | **1.42** | **.009** | |  | | 0.41 | 0.74 | 1.50 | .581 | |  | | -0.08 | 0.13 | .523 | |
|  | Self-harm * Informal support | **-0.77** | **0.09** | **0.46** | **<.001** | |  | | -0.42 | 0.53 | 0.66 | .435 | |  | | <0.01 | 0.09 | .998 | |
|  | Self-harm * Online support | ***0.40*** | ***0.15*** | ***1.49*** | ***.009*** | |  | | **1.99** | **0.98** | **7.34** | **.042** | |  | | 0.05 | 0.18 | .797 | |
|  | Neurodivergence * Informal support | **-0.27** | **0.11** | **0.76** | **.011** | |  | | -0.57 | 0.64 | 0.57 | .377 | |  | | -0.09 | 0.11 | .412 | |
|  | Neurodivergence * Online support | -0.02 | 0.19 | 0.98 | .930 | |  | | 1.54 | 1.30 | 4.66 | .237 | |  | | 0.19 | 0.22 | .401 | |
|  | Self-harm * Neurodivergence * Informal support | -0.01 | 0.13 | 0.99 | .938 | |  | | <0.01 | 0.74 | 1.00 | .996 | |  | | 0.19 | 0.14 | .163 | |
|  | Self-harm * Neurodivergence * Online support | -0.20 | 0.22 | 0.82 | .369 | |  | | -2.34 | 1.46 | 0.10 | .109 | |  | | -0.13 | 0.25 | .597 | |
| Model Fit | |  |  |  |  | |  | |  |  |  |  | |  | |  |  |  | |
|  | Marginal R^2^ | .12 |  |  |  | |  | | .01 |  |  |  | |  | | .09 |  |  | |
|  | Conditional R^2^ | .38 |  |  |  | |  | | .95 |  |  |  | |  | | .46 |  |  | |
| *n* | |  |  |  |  | |  | |  |  |  |  | |  | |  |  |  | |
|  | Participants | 4258 |  |  |  | |  | | 2109 |  |  |  | |  | | 1935 |  |  | |
|  | Observations | 85160 |  |  |  | |  | | 5461 |  |  |  | |  | | 4743 |  |  | |

*Note.* Formal support was used as a refence category for both Informal and Online support effects. Lines in **‘Bold’**, indicate significant effects. Lines in *‘Italics’*, indicates the effects that changed significance in comparison to the main analysis.
